# The epithelial cell response to health and disease associated oral biofilm models

**DOI:** 10.1111/jre.12395

**Published:** 2016-06-22

**Authors:** G. Ramage, D. F. Lappin, E. Millhouse, J. Malcolm, A. Jose, J. Yang, D. J. Bradshaw, J. R. Pratten, S. Culshaw

**Affiliations:** ^1^ Oral Sciences Research GroupGlasgow Dental SchoolSchool of MedicineUniversity of GlasgowGlasgowUK; ^2^ Institute of Infection, Immunity and InflammationCollege of Medical, Veterinary and Life SciencesUniversity of GlasgowGlasgowUK; ^3^ GlaxoSmithKline Consumer HealthcareSt George's AvenueWeybridgeSurreyUK

**Keywords:** biofilm, epithelial, inflammation

## Abstract

**Background and Objective:**

Different bacteria differentially stimulate epithelial cells. Biofilm composition and viability are likely to influence the epithelial response. *In vitro* model systems are commonly used to investigate periodontitis‐associated bacteria and their interactions with the host; therefore, understanding factors that influence biofilm–cell interactions is essential. The present study aimed to develop *in vitro* monospecies and multispecies biofilms and investigate the epithelial response to these biofilms.

**Material and Methods:**

Bacterial biofilms were cultured *in vitro* and then either live or methanol‐fixed biofilms were co‐cultured with epithelial cells. Changes in epithelial cell viability, gene expression and cytokine content of culture supernatants were evaluated.

**Results:**

Bacterial viability was better preserved within mixed‐species biofilm culture than within single‐species biofilm culture. Both mixed‐ and single‐species biofilms stimulated increased expression of mRNA for interleukin 8 (*IL8*), C‐X‐C motif chemokine ligand 3 (*CXCL3*), C‐X‐C motif chemokine ligand 1 (*CXCL1*), interleukin 1 (*IL1*), interleukin 6 (*IL6*), colony‐stimulating factor 2 (*CSF2*) and tumour necrosis factor (*TNF*), and the response was greatest in response to mixed‐species biofilms. Following co‐culture, cytokines detected in the supernatants included IL‐8, IL‐6, granulocyte colony‐stimulating factor and granulocyte–macrophage colony‐stimulating factor, with the greatest release of cytokines found following co‐culture with methanol‐fixed, mixed‐species biofilms.

**Conclusions:**

These data show that epithelial cells generate a distinct cytokine gene‐ and protein‐expression signature in response to live or fixed, single‐ or multispecies biofilms.

The gingival sulcus is lined by a nonkeratinized, stratified squamous epithelium that is in constant contact with bacteria and their products. As such, this epithelial barrier is integral to the maintenance of oral health and immune homeostasis 1. The epithelium provides a physical barrier, as well as playing an active role in innate host defence by releasing soluble mediators such as cytokines 2. Advances in our understanding of the microbiology of periodontal disease have revealed the complexity of the biofilm. Key species, such as *Porphryomonas gingivalis*, are instrumental in biofilm dysbiosis but depend on commensals with capability as accessory pathogens, such as *Streptococci* spp. 3. *In vivo*, oral bacteria, such as *P. gingivalis*, are found only in multispecies biofilms within the oral cavity. Numerous bacteria in the oral biofilm have synergistic or antagonistic interactions, which can shape the oral biofilm, and these bacterial interactions are likely to impact on host–bacteria interactions. The host immune response to the biofilms plays a key role in periodontal disease pathogenesis. Therefore, investigating the interactions between oral bacteria and the host immune system is paramount to understanding the aetiology of periodontal disease. Historically, many *in vitro* studies of the host–pathogen relationship in the oral cavity investigated bacteria‐derived soluble or secreted molecules, such as lipopolysaccharide or proteases, or used planktonic single species (which could be viable, fixed or heat inactivated) co‐cultured with human primary cells or cell lines. These studies identified the specific role of molecules, receptors and ligands, as well as the response patterns to specific bacteria 4. Given the close proximity of the oral biofilm to the oral epithelial surface, their interaction is of particular interest, particularly as epithelial cells are capable of myriad functions and of regulating the subsequent inflammatory response 5. Among their myriad findings, these studies revealed that challenging human gingival epithelial cells with live or heat‐killed ‘early colonizer’ bacteria, such as *Streptococcus gordonii*, in planktonic form, resulted in the release of minimal amounts of cytokines, such as interleukin (IL)‐6, IL‐8 and IL‐1β. In contrast, cytokine release was significantly elevated in response to disease‐associated species such as *Fusobacterium nucleatum* 6, 7. Co‐culture studies of *P. gingivalis* and epithelial cells demonstrated that *P. gingivalis* degrades cytokines and invades host cells 8, 9. *In vivo*, in the mouth, bacteria exist as complex multispecies biofilms, and therefore *in vitro* studies have increasingly sought to reproduce the complexities of these host–biofilm interactions 10. Different studies have investigated the effects on mammalian cells of live and dead bacteria, bacteria in planktonic and biofilm forms, single species of bacteria and multiple species of bacteria in various combinations. In the present study we sought to compare the epithelial cell responses to different bacteria, as single and multispecies biofilms, to build a comprehensive picture of cellular responses.

## Material and methods

### Bacteria and biofilms

Bacteria and biofilms were prepared as previously described 11. Briefly, *P. gingivalis* ATCC 33277 and *F. nucleatum* ATCC 10596 were grown at 37°C in Schaedler Anaerobe Broth (Oxoid, Cambridge, UK) for 2 d in an anaerobic chamber (85% N_2_, 10% CO_2_ and 5% H_2_; Don Whitley Scientific Limited, Shipley, UK). *Aggregatibacter actinomycetemcomitans* ATCC 43718 and *Streptococcus mitis* ATCC 12261 were grown at 37°C in tryptic soy broth (Sigma, Poole, UK), supplemented with 0.8% weight by volume (w/v) glucose (BDH, Poole, UK) and 0.6% (w/v) yeast extract (Oxoid, Cambridge, UK), for 24 h in 5% CO_2_. The bacteria were washed with phosphate‐buffered saline then standardized to approximately 1 × 10^7^ colony‐forming units/mL in artificial saliva (AS) containing porcine stomach mucins (0.25%, w/v) (Sigma‐Aldrich, UK), sodium chloride (0.35%, w/v) (VWR, Leuven, Belgium), potassium chloride (0.02%, w/v) (VWR), calcium chloride dihydrate (0.02%, w/v) (VWR), yeast extract (0.2%, w/v) (Formedium, Hunstanton, UK), Lab‐Lemco powder (0.1%, w/v) (Oxoid, Hampshire, UK) and Proteose‐Peptone (0.5%, w/v) (Sigma‐Aldrich) in ddH_2_O (Thermo Scientific). Urea (Sigma‐Aldrich) was diluted in ddH_2_O [to give a stock solution of 40% (w/v) urea] and added to a final concentration of 0.05% (v/v) in AS.

Biofilms were prepared as previously described 11. Briefly, for monospecies *S. mitis* biofilms, 500 μL of standardized *S. mitis* in AS was transferred to 24‐well plates (Corning), containing Thermanox^™^ coverslips (13 mm diameter; Fisher Scientific, Loughborough, UK), then incubated at 37°C in 5% CO_2_ for 48 h. *Porphyromonas gingivalis* was prepared similarly but incubated at 37°C in an anaerobic environment for 96 h. For multispecies biofilms, *S. mitis* in AS was added for the first 24 h, at 37°C, 5% CO_2;_ supernatant was then removed and *F. nucleatum* in AS was added and the biofilms were incubated anaerobically for a further 24 h. The supernatant was removed and finally the standardized *P. gingivalis* and *A. actinomycetemcomitans* in AS were added to the biofilm and incubated at 37°C in the anaerobic chamber for a further 4 d. In all cases the AS was replaced daily. Biofilms were visualized by scanning electron microscopy, as previously described 11. Briefly, biofilms were washed three times in sterile phosphate‐buffered saline, then fixed and viewed using a JEOL JSM‐6400 scanning electron microscope (Herts, UK). Biofilms or bacteria described as ‘dead’ or ‘fixed’ were fixed in 100% methanol.

### Epithelial cell co‐culture

OKF6/TERT2 cells (gifted by the Rheinwald Laboratory; Brigham and Women's Hospital, Boston, MA, USA), an immortalized human oral keratinocyte cell line, were cultured with biofilms or planktonic bacteria as previously described 11 and as indicated in the figure legends. Each experiment was carried out using an independently grown ‘batch’ of biofilms, cultured in triplicate in wells with epithelial cells, and all experiments were repeated at least twice.

### Epithelial cell gene‐expression analysis

RNA extraction was performed using the RNeasy Mini Kit (Qiagen, Hilden, Germany), according to the manufacturer's instructions. A NanoDrop 1000 spectrophotometer (Thermo Scientific) was used to assess RNA concentration and quality. Five‐hundred nanograms of RNA was reverse transcribed, using ‘high capacity RNA‐to‐cDNA’ kits (Applied Biosystems, Foster City, CA, USA), according to the manufacturer's instructions. Gene‐expression analysis was carried out using a custom‐designed ABI microfluidic Taqman^®^ Low Density Array (Applied Biosystems), which incorporated primer/probe sets to evaluate expression of genes associated with gingivitis 12: colony‐stimulating factor 3 (*CSF3*), interleukin 8 (*IL8*), interleukin‐1alpha (*IL1α*), C‐C motif chemokine ligand 5 (*CCL5*), interleukin‐1beta (*IL1β*), C‐X‐C motif chemokine ligand 3 (*CXCL3*), C‐C motif chemokine ligand 3 (*CCL3*), C‐X3‐C motif chemokine receptor 3 (*CX3CR1*), C‐C motif chemokine ligand 4 (*CCL4*), C‐X‐C motif chemokine ligand 10 (*CXCL10*), C‐X‐C motif chemokine ligand 11 (*CXCL11*), tumour necrosis factor, alpha (*TNFα*), colony‐stimulating factor 2 (*CSF2*), C‐X‐C motif chemokine ligand 1 (*CXCL1*), interleukin 6 (*IL6*) and C‐X‐C motif chemokine ligand 5 (*CXCL5*). Two housekeeping, control genes – TATAA‐box binding protein and glyceraldehyde‐3‐phosphate dehydrogenase (*GAPDH*) – were utilized to span the relative abundance/cycle threshold (*C*_t_) range of the genes on the card. For the gene‐expression analysis the geometric mean of the housekeeping gene *C*_t_ values was subtracted from the target gene values and the ΔΔ*C*_t_ values for each target mRNA were obtained and used in subsequent statistical analysis 13.

### Protein release from epithelial cells

Supernatants harvested from OKF6 epithelial cells, 4 and 24 h after bacterial biofilm challenge, were evaluated for IL‐1α, IL‐6, IL‐8, granulocyte–macrophage colony‐stimulating factor and granulocyte colony‐stimulating factor using Luminex^®^ multiplex beads (ThermoFisher) according to the manufacturer's instructions; and for growth‐regulated alpha protein (Gro‐α, encoded by *CXCL1*), C‐X‐C motif chemokine 10 [CXCL10, also known as interferon‐gamma‐inducible protein (IP‐10)] and C‐C motif chemokine 5 (CCL5; also known as RANTES) using ELISA (Peprotech, London, UK) according to the manufacturer's instructions.

### Epithelial cell viability

The viability of epithelial cells was analysed using alamarBlue^®^ dye (ThermoFisher), according to the manufacturer's instructions. The cell culture medium was removed from the epithelial monolayer and epithelial cells were washed and then incubated with 10% alamarBlue^®^. Cell viability was assessed using alamarBlue^®^ and data are expressed as percentage of the difference between the reductions of intensity of alamarBlue^®^ in treated cells versus untreated controls. DNA and histone release were also evaluated to assess cell death, using the Cell Death Detection ELISA^PLUS^ (Roche Applied Science, Mannheim, Germany) according to manufacturer's instructions. The specific enrichment of mono‐ and oligonucleosomes released into the cytoplasm was assessed and the ratio between the absorbance values obtained in media control and biofilm treated epithelial cells calculated. Epithelial cells treated with 4 μg/mL of camptothecin served as the positive control.

### Statistical analysis

Graph production, data distribution and statistical analysis were performed using graphpad prism (version 6; Graphpad Software Inc., La Jolla, CA, USA), microsoft excel and paleontological statistics (past; v3.02) software 14. After assessing whether data conformed to a normal distribution before and after data transformations, ANOVA and *t*‐tests were used to investigate significant differences between independent groups of data that approximated to a Gaussian distribution. Welch's *t*‐test was used when there were significant differences in the variance of data between the groups. Although the analysis was principally exploratory in nature, a Bonferroni correction was applied to account for multiple comparisons of the data. Log‐transformed data were utilized to carry out principal component analysis using past.

## Results

### Biofilm growth *in vitro*

**Figure 1:**
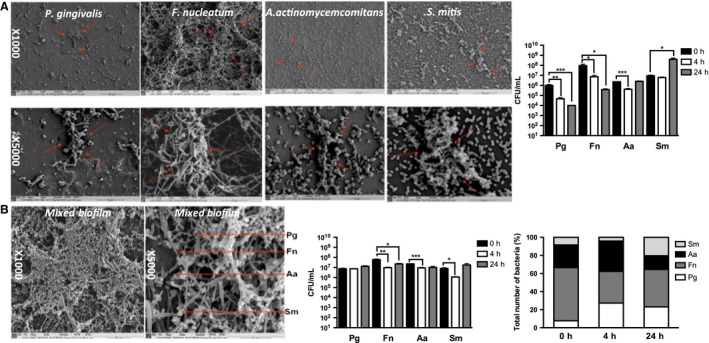
Scanning electron micrographs and total viable cell counts of fresh monospecies and multispecies biofilms. (A) Scanning electron microscopy images of *Porphryomonas gingivalis* (Pg), *Fusobacterium nucleatum* (Fn), *Aggregatibacter actinomycetemcomitans* (Aa) and *Streptococcus mitis* (Sm) monospecies biofilms, and their survival over 24 h in cell‐culture medium. The bar chart shows the mean number [given in colony‐forming units (CFU)/mL] of viable bacteria recovered from the biofilms ± standard error of the mean. (B) Scanning electron microscopy images of multispecies biofilms, arrows in the SEM show examples of each of the 4 different bacteria. Data shown represent survival over 24 h in cell‐culture medium. The results are given in CFU/ml (grouped bar chart) and as proportional changes in biofilm composition shown as percentage total (%) of the total number of bacteria mixed‐species biofilm (stacked bar chart). Statistical analysis was performed on square root transformations of the CFU/mL value using a two‐tailed independent‐sample *t*‐test (*p < 0.01, **p < 0.001, ***p < 0.0001).

*Porphryomonas gingivalis*,* S. mitis*,* F. nucleatum* and *A. actinomycetemcomitans* each individually formed communities of bacteria that adhered to a hydroxyapatite disk (Fig. 1A). These were of variable architecture: *F. nucleatum* formed multilayered, relatively dense networks of bacteria; *P. gingivalis* formed relatively sparse groups of bacteria; and the mixed‐species biofilms formed a dense, complex multilayered structure, which was notably more substantial than any of the single‐species biofilms (Fig. 1B). The established *S. mitis* biofilms continued to grow in cell‐culture conditions for 24 h. Over 24 h, A. *actinomycetemcomitans* maintained constant viability; the viability of *F. nucleatum* and *P. gingivalis* significantly decreased (Fig. 1A). The proportions of bacteria in the mixed‐species biofilms changed following culture in cell‐culture conditions, with a reduction in the proportions of *F. nucleatum* and *A. actinomycetemcomitans*, and increases in the proportions of *P. gingivalis* and *S. mitis* (Fig. 1B).

### Expression of genes by epithelial cells following co‐culture with different bacterial biofilms

**Figure 2:**
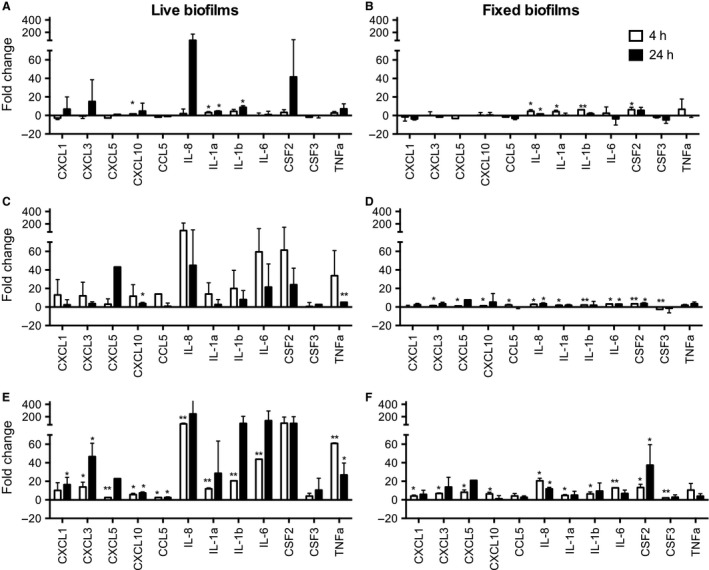
Gene‐expression changes in epithelial cells following stimulation with different biofilms. OKF6‐TERT2 epithelial cells were challenged with live (A, C and E) or fixed (B, D and F) biofilms of *Streptococcus mitis* (A and B), *Porphryomonas gingivalis* (C and D) and mixed‐species biofilms (E and F) for 4 h (white bars) and 24 h (black bars). Expression of mRNA for gingivitis related genes was assessed using the TaqMan^®^ Low Density Array. Gene expression was normalized to that of the glyceraldehyde‐3‐phosphate dehydrogenase (*GAPDH*) endogenous control. The bars in each chart represent fold change in gene expression relative to the medium‐only control. Statistical analyses were performed on ΔΔ*C*_t_ values. *Significantly different from the medium‐only control, *p* < 0.05; **significantly different from the medium‐only control after Bonferroni correction of the *p* value. Gene symbols and definitions: *CCL5*, C‐C motif chemokine ligand 5; *CSF2*, colony‐stimulating factor 2; *CSF3*, colony‐stimulating factor 3; *CXCL1*, C‐X‐C motif chemokine ligand 1; *CXCL3*, C‐X‐C motif chemokine ligand 3; *CXCL5*, C‐X‐C motif chemokine ligand 5; *CXCL10*, C‐X‐C motif chemokine ligand 10; *IL1α*, interleukin‐1alpha; *IL1β*, interleukin‐1beta; *IL8*, interleukin 8; *TNFα*, tumour necrosis factor, alpha.

**Figure 3:**
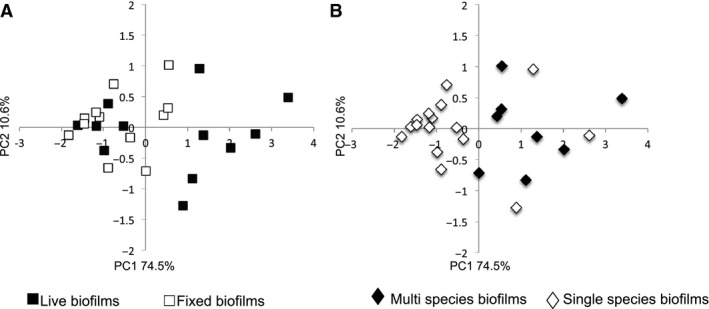
Principal component analysis of changes in epithelial cell gene expression following exposure to different biofilms. The data depicted in Fig. 2 were subjected to principal component analysis. Each point represents all the experiments in which cells were stimulated by exposure to a particular condition and is a vector positioned on each axis according to the percentage variance from the origin, on two principal component axes (PC1 and PC2). (A) Data annotated to compare the response to live biofilms (solid squares) with the response to fixed biofilms (open squares). (B) Data annotated to compare the response to mixed‐species biofilms (solid diamonds) with the response to single‐species (open diamonds) biofilms.

Epithelial cells were cultured alone (media control) or with single‐species or multispecies bacterial biofilms suspended on a disc placed 0.5 mm above the epithelial cells. This system allows culture of biofilms of live bacteria with adjacent fluid and is reminiscent of the gingival crevicular fluid flow in the periodontal pocket 15. Thus, the epithelial cells encounter a small number of bacteria that are shed from the biofilm and are also exposed to products of the live bacteria in the biofilm. The bacteria in the periodontal pocket, in particular *P. gingivalis*, are known to generate products that are cytotoxic and that can degrade cytokines. Therefore, to establish the extent to which live bacteria and their products contribute to the host response, cells were stimulated with both fixed and live biofilms. The *S. mitis* and *P. gingivalis* monospecies biofilms were selected as exemplar monospecies biofilms, of commensal‐associated and disease‐associated bacteria, respectively, and the mixed four‐species biofilm was used as an example of a more complex multispecies community. The epithelial cell responses were investigated after 4 or 24 h of culture with bacteria, and all experiments were repeated at least twice. Comparisons of the fold change in gene expression compared with media control showed that live bacterial biofilms (*P. gingivalis*,* S. mitis* or mixed species) stimulated greater changes in gene expression than did methanol‐fixed bacterial biofilms. There was a progressive increase in both the number of genes up‐regulated and the magnitude of increase from *S. mitis‐*stimulated epithelial cells, to *P. gingivalis*‐stimulated cells, with mixed biofilms resulting in the greatest qualitative and quantitative increases in gene expression (Fig. 2). In response to stimulation with mixed‐species biofilms there was notable increase in the expression of mRNA for *IL8*,* CXCL3*,* CXCL1, IL1*,* IL6*,* CSF2* and *TNF*α. In general, live biofilms stimulated greater changes in gene expression than did fixed biofilms (Fig. 2 and Table 1). Principal component analysis of the gene‐expression data for all genes and all conditions was carried out (Fig. 3). Each point represents a vector derived for each experimental condition from the first two principal components, which accounted for 74.5% and 10.6% of the variance, respectively. Visual representation of these data shows clustering of the epithelial cell response to live biofilms compared with fixed biofilms (Fig. 3A), and of the epithelial cell response to mixed‐species biofilms compared with monospecies biofilms (Fig. 3B).

**Table 1:** Comparison of gene expression (mRNA levels), at the 4‐h culture time point

Gene	smbflive	smbflive	smbfdead	smbfdead	smbfdead	pgbfdead	pgbfdead	mix live
	mix live	mix dead	pgbfdead	mix live	mix dead	mix live	mix dead	mix dead
*CXCL1*	0.082	**0.019**	0.766	0.143	0.139	0.125	**0.045**	0.398
*CXCL3*	**0.023**	**0.021**	0.946	0.088	0.147	**0.025**	**0.016**	0.142
*CXCL5*	**0.019**	**0.015**	0.054	**0.015**	**0.014**	*0.060*	**0.020**	**0.041**
*CXCL10*	**0.032**	**0.036**	0.940	0.128	0.117	**0.022**	**0.026**	0.723
*CCL5*	**0.047**	0.085	0.061	**0.006**	0.093	*0.651*	0.379	0.445
*IL8*	**0.042**	0.112	0.281	**0.009**	**0.031**	**0.002** *	**0.006**	**0.010**
*IL1α*	**0.033**	0.267	0.138	0.051	0.688	**0.009**	0.055	**0.023**
*IL1β*	*0.122*	0.428	**0.002** *	**0.001** *	0.961	***0.004***	0.052	*0.113*
*IL6*	*0.062*	**0.024**	0.836	*0.210*	*0.356*	***0.011***	**0.001** *	***0.008***
*CSF2*	0.116	0.142	*0.340*	0.147	0.157	0.102	**0.022**	0.245
*CSF3*	0.140	**0.003** *	0.364	0.155	**0.028**	0.124	**0.001** *	0.475
*TNF*	*0.072*	0.200	0.608	*0.259*	0.636	***0.042***	0.132	*0.165*

Values calculated using Welch's *t*‐test are shown in italics. Bold denotes statistically significant differences in the comparison listed at top.

bf, biofilm; dead, fixed; mix, mixed species; pg, *Porphyromonas gingivalis*; sm, *Streptococcus mitis*. Gene symbols and definitions: *CCL5*, C‐C motif chemokine ligand 5; *CSF2*, colony‐stimulating factor 2; *CSF3*, colony‐stimulating factor 3; *CXCL1*, C‐X‐C motif chemokine ligand 1; *CXCL3*, C‐X‐C motif chemokine ligand 3; *CXCL5*, C‐X‐C motif chemokine ligand 5; *CXCL10*, C‐X‐C motif chemokine ligand 10; *IL1α*, interleukin‐1alpha; *IL1β*, interleukin‐1beta; *IL8*, interleukin 8; *TNF*, tumour necrosis factor.

*Significant after Bonferroni correction. If comparisons showed no differences these were omitted from the table.

### Production of cytokines from epithelial cells co‐cultured with bacteria

**Figure 4:**
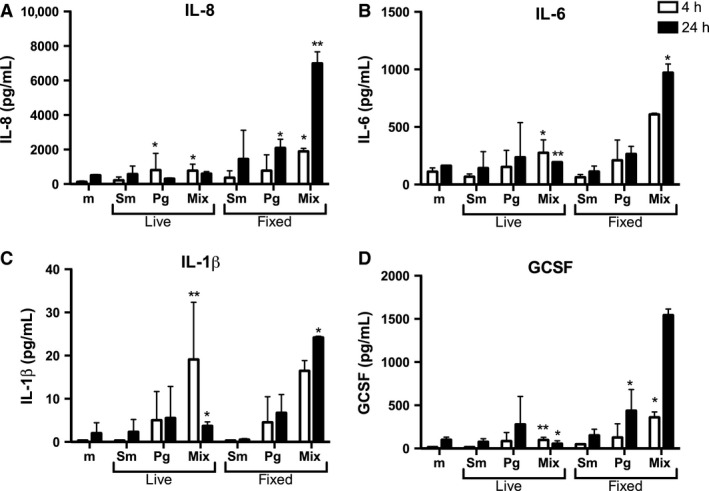
Epithelial cell cytokine release following stimulation with different biofilms. OKF6‐TERT2 epithelial cells were cultured with medium only (m) or challenged with live or fixed biofilms of *Streptococcus mitis* (Sm), *Porphryomonas gingivalis* (Pg) and mixed‐species (Mix) biofilms for 4 h (white bars) and 24 h (black bars). Protein concentrations in the cell‐culture supernatants were measured using Luminex® multiplex beads. (A) IL‐8, (B) IL‐6, (C) IL‐1β, (D) GCSF. Each bar represents the mean ± standard error of the mean of duplicate measurements of two independent experiments. *Significantly different from medium‐only control; *p* < 0.05. **Significantly different from the medium‐only control after Bonferroni correction of the *p* value. G‐CSF, granulocyte colony‐stimulating factor; IL‐1β, interleukin‐1β; IL‐6, interleukin‐6; IL‐8, interleukin‐8.

Following the investigation of changes in gene expression, we sought to determine changes in release of cytokines into the cell‐culture supernatant. Live single‐species and mixed‐species biofilms stimulated modest release of IL‐8, IL‐6 and granulocyte colony‐stimulating factor. Compared with the medium‐only control, the mixed live biofilms stimulated significant cytokine release at both 4 and 24 h (Fig. 4, and summary of statistical analysis in Table 2).

**Table 2:** Comparison of cytokine concentrations detected in cell‐culture supernatants

A: cytokines assessed at 4‐h culture time point	smbflive	smbfdead	smbfdead	mix live
	mix live	mix live	mix dead	mix dead
IL‐8	**00.078**	0.174	0.404	0.118
IL‐1β	**0.001**	**0.001** *	**0.036** *	0.968
IL‐6	0.071	**0.014**	0.069	*0.216*
GM‐CSF	**0.033**	**0.033**	0.500	0.359
G‐CSF	**0.002** *	**0.004**	0.094	**0.034** *
TNF	1.000	1.000	1.000	1.000

B: cytokines assessed at 24‐h culture time point	smbflive	smbflive	smbfdead	smbfdead	pgbflive	pgbflive	pgbfdead	pgbfdead	mix live
	pgbfdead	mix live	mix live	mix dead	pgbfdead	mix live	mix live	mix dead	mix dead
IL‐8	0.162	0.055	0.202	0.772	**0.010**	**0.001** *	**0.022**	**0.030**	**0.004**
IL‐1β	0.348	0.083	**0.002** *	**0.025**	0.663	*0.337*	*0.199*	0.430	**0.007**
IL‐6	0.400	*0.231*	**0.020**	0.198	0.598	*0.368*	**0.020**	0.241	**0.001** *
GM‐CSF	**0.009**	**0.000** *	**0.015**	0.423	0.357	*0.241*	**0.026**	**0.009**	**0.000** *
G‐CSF	0.087	**0.013**	**0.020**	0.205	0.532	*0.299*	0.085	0.077	**0.017**
TNF	1.000	0.423	0.423	1.000	1.000	0.423	1.000	1.000	0.423

G‐CSF, granulocyte colony‐stimulating factor; GM‐CSF, granulocyte–macrophage colony‐stimulating factor; IL‐1α, interleukin‐1alpha; IL‐1β, interleukin‐1beta; IL‐8, interleukin‐8; TNF, tumour necrosis factor.

Values calculated using Welch's *t*‐test are shown in italics. Bold denotes statistically significant differences in the comparison listed at top.

bf, biofilm; dead, fixed; mix, mixed species; pg, *Porphyromonas gingivalis*; sm, *Streptococcus mitis*. Comparisons that showed no differences were omitted from the table.

*Significant after Bonferroni correction.

### Epithelial cell viability following co‐culture with biofilms

**Figure 5:**
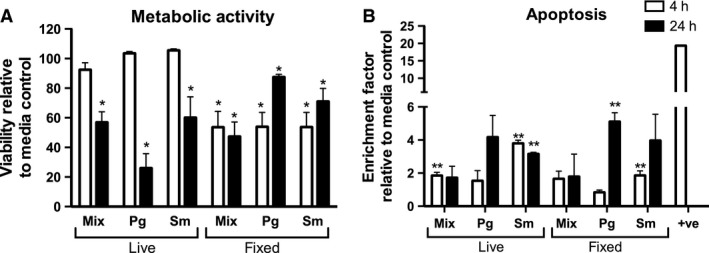
Survival of epithelial cells after challenge with bacterial biofilms. Epithelial cells were challenged with live or methanol‐fixed mixed species (Mix), *Porphryomonas gingivalis* (Pg) or *Streptococcus mitis* (Sm) biofilms for 4 h (white bars) or 24 h (black bars). (A) Cell viability was assessed by staining with alamarBlue^®^. Data are expressed as percentage viability relative to untreated control cells; bars represent mean ± standard error of the mean of triplicate measurements of two independent experiments. (B) Epithelial cell apoptosis was assessed by histone ELISA. Bars represent mean ± standard error of the mean of two independent wells in two independent experiments. Statistical analysis was performed on square root transformation of the data and consisted of the *t*‐test or Welch's *t*‐test after assessing the data variance. *p < 0.05, less than the medium‐only control in regard to alamarBlue^®^ reduction. For histone measurements, all treatments were significantly lower than the positive control (*p* < 0.01). **p < 0.05, greater than the medium‐only control.

To investigate changes in cell viability, OKF6‐TERT2 epithelial cells were challenged with live or methanol‐fixed, mixed or single‐species biofilms, for 4 and 24 h. Compared with the viability of cells cultured with medium only, the majority of the biofilms caused a statistically significant reduction in cell viability, as measured by either method (Table 3). The magnitude of change in viability, assessed by alamarBlue^®^, varied, with epithelial cells challenged with live biofilms appearing to maintain viability close to that of medium control for 4 h, and viability declining at 24 h (Fig. 5A). All biofilms caused statistically significantly elevated release of histone compared with the medium control (Fig. 5B).

**Table 3:** Viability of cells stimulated for 4 and 24 h with different biofilms, compared with the medium‐only control. Values were obtained using multiple comparisions by ANOVA with post‐hoc *t*‐tests

Study time point and method used to determine cell viability	mix live	pg bf live	sm bf live	mix dead	pg bf dead	sm bf dead
4 h						
AlamarBlue^®^	0.086	**1.000**	**1.000**	**0.001**	**0.000**	**0.018**
Histone	**0.0025** *	**0.008**	**0.003**	**0.006**	**0.0016** *	**0.004**
24 h						
AlamarBlue^®^	**0.004**	**0.0025** *	**0.003**	**0.0015** *	**0.0004** *	**0.011**
Histone	**0.0004** *	**0.019**	**0.0013** *	**0.017**	**0.008**	**0.023**

bf, biofilm; dead, fixed; mix, mixed species; pg, *Porphyromonas gingivalis*; sm, *Streptococcus mitis*.

Bold denotes statistically significant differences compared with medium control.

*Significant after Bonferroni correction.

## Discussion

The data obtained in the present study demonstrate that an epithelial cell line generates a distinct cytokine gene‐ and protein‐expression signature in response to live or dead, single or multispecies biofilms. The data imply immune functional consequences, for both the host and bacteria, of differing biofilm composition. The immune response of the epithelial cells appears to be dependent on the type of bacterial challenge. The PCA results demonstrate that mixed biofilms elicit a distinct response compared with single‐species biofilms, and that biofilm viability impacts on the response. Previous studies using bacteria in planktonic culture or a single‐species biofilm of *Streptococcus oralis*,* F. nucleatum* or *A. actinomycetemcomitans* demonstrated species‐specific responses in oral epithelial cells. Generally, planktonic bacteria of greater pathogenicity stimulate increased levels of pro‐inflammatory cytokines, such as IL‐6, IL‐8 and IL‐1β 7, 16. The cells generated a range of cytokines and chemokines, with functions including chemoattraction, promotion of cell survival, endothelial cell activation and increased adhesion molecule expression and stimulation of cytokine production by other cell types 7, 16. Thus, biofilms, in particular the mixed‐species biofilm, stimulate all the hallmark cytokines of gingival inflammation and can activate epithelial cells to coordinate many features of gingival inflammation. The maturation state of the biofilm has been shown to result in differential expression of IL‐8 by epithelial cells, with mature biofilms being more proinflammatory than less‐complex biofilms 17. The relatively modest response to the *S. mitis* biofilms, observed in these studies, is consistent with previous observations that show a similar epithelial cell response to monospecies *Streptococcus* biofilms or multispecies biofilms containing bacteria classed as ‘early colonizers’ 7, 16, 18. Our data show clear discrepancies between the levels of gene expression and the levels of protein released, particularly in response to live mixed‐species biofilm, which caused statistically significantly more up‐regulation of mRNA for IL‐6, IL‐8, IL‐1 and CXCL5 than the fixed biofilms, but only relatively small increases in concentration of cytokines in supernatants. We speculate that this enhanced response to live cells reflects stimulation by soluble products released from viable, but not fixed, biofilms. In addition, the biofilm fixation process may have altered bacterial antigens such that they are less stimulatory to epithelial cells.

Higher concentrations of cytokines were detectable in supernatants after culture with dead biofilms than after culture with live biofilms. The epithelial cells clearly respond to these biofilms but there is also likely to be post‐translational modification of cytokines where there is live biofilm present. Studies using multispecies oral biofilm models have reported similar findings when investigating protein expression and attributed this to cytokine degradation by *P. gingivalis*, with reduction of IL‐8 in supernatant following co‐culture only when *P. gingivalis* and their gingipains were present in the biofilm 9, 18, 19. In addition to the impact of specific gene products, there are likely to be effects resulting from strain variation in the individual species within the biofilm. The species chosen in these studies reflected those used in previous studies 9. *Porphyromonas gingivalis* ATCC33277 has type I FimA, expresses gingipains and does not have a capsule. This strain is capable of *in vitro* biofilm formation and will induce alveolar bone loss in animal models 20. *Aggregatibacter actinomycetemcomitans* 43718 is serotype b, which produces cytotoxic membrane vesicles and is clinically associated with aggressive periodontitis 21. Although the coexistence of periodontal microbiota in clusters is well established, there is limited understanding of how different strains coexist. It would be of interest to define how strain variance dictates biofilm characteristics *in vivo* and how this impacts on the host response.

Mixed‐species biofilms had a marked impact on cell metabolic activity and cell death by apoptosis. Similar patterns of cell death were observed using each method, suggesting that the epithelial cell death following exposure to multispecies biofilms is the result of a combination of apoptosis and necrosis. Guggenheim *et al*. 19 observed that human gingival epithelial cells co‐cultured with their ‘subgingival’ nine‐species biofilm model underwent apoptosis in a time‐dependent manner at 4 and 24 h. Studies using gingival tissue biopsies have shown increased levels of apoptosis in periodontitis samples compared with healthy controls, suggesting that tissue destruction by apoptosis plays a role in the pathogenesis of periodontitis 22. Nonetheless, even though there is cell death, the remaining viable cells clearly respond to the biofilms. It is not clear if there are a priori biological differences between the cells that maintain viability and those that die. The increased cell death over time hints that this may be simply a feature of the kinetics of cell exposure to the insult.

In summary, our data show that biofilms differentially modulate the epithelial cell immune response based on biofilm composition. The detailed characterization of the plethora of *in vitro* model systems investigating host–pathogen interactions should yield a picture of the caveats and benefits of different platforms for different applications. Using these data, platforms can be appropriately selected for a variety of host tissues in co‐culture with biofilms and aid in *in vitro* studies carried out to understand disease pathogenesis and to identify potential novel therapeutic targets for periodontal disease.

## Competing interests

EM, JM, DFL, SC and GR have no competing interests. AJ, DB and JP are all employees of GlaxoSmithKline, the sponsor of the study.

## Author's contributions

EM, JM and AJ participated in the study design, carried out the experimental studies on biofilms, performed statistical analysis and were responsible for the manuscript. DFL participated in the study design, statistical analysis and helped draft the manuscript. JY, DB and JP contributed to the study design and supervized manuscript writing. GR and SC conceived the study, participated in the study design and data analysis and were responsible for writing and submission of the final manuscript. All authors read and approved the manuscript.
